# Circadian-clock driven cone-like photoreceptor phagocytosis in the neural retina leucine zipper gene knockout mouse

**Published:** 2010-12-28

**Authors:** Arthur Krigel, Marie-Paule Felder-Schmittbuhl, David Hicks

**Affiliations:** Département de Neurobiologie des Rythmes, CNRS UPR 3212, Institut des Neurosciences Cellulaires et Intégratives, Strasbourg, France

## Abstract

**Purpose:**

Whereas much information is available on rod outer segment phagocytosis by the retinal pigmented epithelium (RPE), corresponding data for cones are quite limited, especially in laboratory models of normal rats and mice with very low cone numbers. To characterize the light and circadian control of cone photoreceptor phagocytosis in mice, we capitalized on the blue cone-like phenotype of neural retina leucine zipper gene (Nrl) null mice (*Nrl^−/−^*).

**Methods:**

*Nrl^−/−^* mice were maintained under standard cyclic light (12h:12h light-dark [LD] cycle; light=300 lux) for one month, then divided into two groups: 1) continued maintenance in LD (36 mice); or 2) transferred to constant darkness (DD; 21 mice) for 36 h. Animals were sacrificed every 3 h over 24 h, and their eyes were rapidly enucleated and fixed. Cryosections were stained using specific cone short-wavelength opsin antibodies. Phagosome numbers in the RPE were quantified with a morphometric system. We monitored the expression of c-mer proto-oncogene tyrosine kinase (MerTK) in wild-type and knockout mice using a specific MerTK antibody.

**Results:**

In LD, cone phagocytosis showed a statistically significant peak of activity 1 h after light onset, 2–3 fold higher than at other times. In constant darkness, the temporal phagocytic profile resembled that of LD (significant peak at 1 h of subjective day), but the number of phagosomes was decreased at all time points. Immunostaining of MerTK in wild-type and *Nrl^−/−^* mice showed expression at the apical surface of the RPE.

**Conclusions:**

Cone-like outer segment phagocytosis in *Nrl^−/−^* mice shows a similar profile to that of rods in normal mice and other species. These data are the first to quantify blue cone-like photoreceptor phagocytosis under different lighting conditions in mice, and suggest this model may constitute a valuable system for investigating circadian regulation of cone function.

## Introduction

Retinal photoreceptors (RP) are composed of two different populations, rods, and cones. Rods are used for nocturnal vision because of their high light sensitivity [1], whereas cones require a relatively high light level to activate them, and are used for diurnal vision [2]. The latter are responsible for color discrimination and high-acuity vision, and are of utmost importance in human vision. In humans and Old World primates, there are three types of cones, containing blue, green, and red light-sensitive pigments (or short wavelength sensitive (S) cones [3], and middle/long wavelength sensitive cones, respectively). In the great majority of mammals, there are only two cone types: a short wavelength-sensitive and a mid/long wavelength-sensitive population [4].

Both rods and cones are known to undergo continuous cyclic turnover, involving the addition of new membranes at the base of the outer segment (OS) and the removal of aged membranes at the distal pole [5]. This removal is achieved by the opposing retinal pigmented epithelium (RPE), which phagocytose and digest the shed OS membranes [6]. In recent years, much progress has been made in identifying molecular components of the phagocytic pathway, including membrane-bound receptors such as MerTK and αVβ5 integrin, and ligands [7-9]. Mutations in the Mertk receptor lead to inherited retinal degeneration in animals [7,10] and humans [11]. The great majority of data have been obtained for rods, in part because conventional laboratory rodent (mouse and rat) retinas are composed of 97% rods and only ~3% cones [12,13]. A genetically modified mouse line has become available for the study of rod differentiation, the neural retina leucine zipper gene (Nrl) knockout mouse (*Nrl*^−/−^) [14]. Deletion of the transcription factor *Nrl* results in the complete absence of rods, as revealed by histology, immunocytochemistry, electrophysiology, and gene expression analysis [14,15]. Morphological, molecular, and electrophysiological features of the *Nrl*^−/−^ photoreceptors seem to be identical to blue or short wavelength light-sensitive S cones [14]. Hence, this retina provides a potential means for the investigation of blue cone function and cone-specific genes [16].

In the present study, we have used homozygous *Nrl*^−/−^ mice to ask whether cone phagocytosis shows daily rhythms, if any such rhythms resemble or differ from those known for rods, and whether these rhythms are maintained under constant darkness. We show that rhythmic phagocytosis does indeed occur, in both cyclic light and constant darkness, and that it resembles rod behavior.

## Methods

### Animals and handling

**Figure 1:**
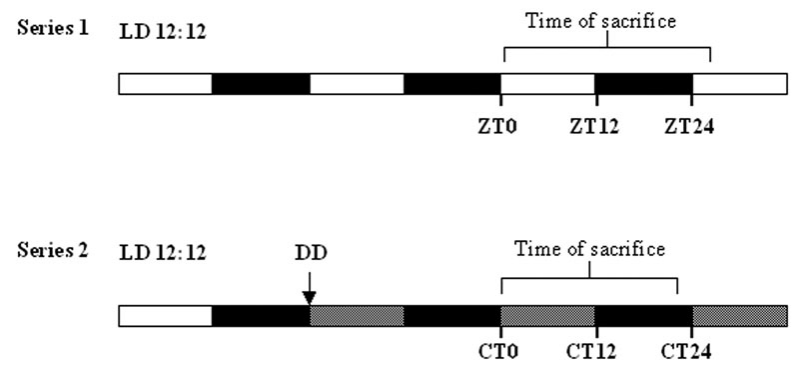
Schematic diagram showing schedule of lighting conditions and sampling points. Arrow in series 2 indicates times at which animals were switched to constant darkness condition. Thin vertical bar joined by a horizontal bar indicates the beginning and end of the sampling period. Series 1 is the control condition, with animals maintained under light-dark conditions (alternating white bars [12 h light=300 lux,] and black bars [12 h dark]) throughout the experiment. Series 2 represents total darkness (alternating black dots on a white background [subjective day] and black bars).

This study was conducted with *Nrl*^−/−^ mice, raised in our animal facilities. The strain was originally obtained from Dr. C. Grimm at the Laboratory of Retinal Cell biology, University Hospital, Switzerland, with permission from Dr. A. Swaroop (NIH, Bethesda, MD). The colony was maintained on a 12 h:12 h light-dark (LD) cycle in an ambient temperature of 22 °C for four weeks. Young adult wild-type C57Bl/6 mice (n=3), the strain on which the knockout line was created, were used as a positive control for antibody binding and specificity. All experiments were conducted according to ethical guidelines in operation at the institute, and adhered to the ARVO Statement for the Use of Animals in Ophthalmic and Vision Research. Two experimental series were made (shown schematically in Figure 1): in the first group, a total of 36 knockout mice were used, composed of mostly young adults (2 months, n=30), with some individuals of 5 months (n=6). Older individuals were spread equally among sample points to eliminate any age-related bias. Animals were euthanized every 3 h during the LD cycle (n=4 for each time point). Lights were turned on at 7 AM, defined as zeitgeber time, hour zero (ZT0). Animals were killed at ZT1, 4, 7, 10, 13, 16, 19, and 22, and at an additional point in the following 24 h cycle (ZT24+1). The second group of animals (n=21) was raised in LD until they reached young adulthood (2 months). The animals were then placed in total darkness (constant darkness [DD]), for 36 h, and therefore experienced a complete cycle of subjective day and night before sample collection. Euthanasia commenced at circadian time (CT) 1 (i.e., 1 h after subjective light onset), and continued every 3 h at CT4, 7, 10, 13, and 16 (n=3 per time point).

In both groups during the night time (i.e., ZT12–24 for LD cycles and permanently for DD), darkness was total (no background dim red light), and euthanasia and enucleation were performed using night vision goggles. For every time point and in both groups, eyes were rapidly removed by dissecting the surrounding ocular muscle and optic nerve. A small hole was made with a 25-gauge hypodermic needle at the level of the ora serrata, and eyeballs were fixed overnight in 4% paraformaldehyde in buffered phosphate saline (0.1 M PBS; l. 91, NaCl 7.34 g/l, KCl 270 mg/l, Na_2_HPO_4_ 10 mg/l, NaH_2_PO_4_ 3 mg/l, pH 7.3), at 4 °C.

### Immunohistochemistry and quantification

Eyes were rinsed in cold PBS for 1 h, then transferred to an ascending series of sucrose solutions (10%, 20%, and 30%, each for 2 h) and embedded in optimal cutting temperature resin (Tissue Tek; Sakura Fintek, Tokyo, Japan), and 10 μm thick cryostat sections were prepared and stored at −20 °C until ready for use for staining. Immunostaining and quantification of phagosomes was performed as published previously by us [17]. Briefly, sections were permeabilized and then saturated with PBS containing 3% BSA, 0.1% Tween-20, and 0.1% sodium azide (buffer A) for 30 min. Sections were incubated overnight at 4 °C with polyclonal anti-mouse S-cone opsin antibody [18] (the generous gift of Dr. Cheryl Craft, Doheny Eye Institute, University of California, Los Angeles, CA) at a ~1 µg/ml final concentration in buffer A. Antibody binding was visualized using secondary fluorescent conjugated anti-rabbit IgG-Alexa 594 (Molecular Probes Ltd., Eugene, OR). Since the *Nrl^−/−^* retina exhibits partial degeneration with a scalloped appearance of the outer nuclear layer and the formation of rosettes [14], quantitative measurements were performed only in areas devoid of rosettes and degenerated cells. Any immunopositive structure with a diameter of ≥1 μm lying within the RPE sub-cellular space (visualized by faint background lighting to show pigmentation) was scored as a phagosome. Counting of phagosomes was performed by aligning a 150×150 μm grid placed within the eyepiece, parallel with the RPE layer [17]. The phagosome counts are expressed as the sum of all four sections per eye.

For comparative studies on phagocytic activity among different species, we also processed frozen sections of adult Wistar rats and adult C57Bl6 mice, from animals killed at ZT1. These sections were only stained with anti-rhodopsin (rho4D2) antibody [19] as described above, and analyzed for rod phagosome numbers using the same morphometric grid procedure.

We also performed immunostaining for MerTK using a rabbit polyclonal MerTK antibody (the generous gift of Dr. G. Lemke, Scripps Institute, La Jolla, CA) [20] to verify the expression and distribution of MerTK in the RPE of *Nrl^−/−^* mice, compared to wild-type mice. Wild-type mouse retinal sections were double-immunolabeled with MerTK and monoclonal anti-rhodopsin antibody Rho-4D2. Antibody binding was detected with anti-rabbit IgG-Alexa 594 and anti-mouse IgG-Alexa 488, following the protocol given above. *Nrl^−/−^* mouse retinal sections were processed differently, to visualize the correspondence of MerTK and cone OS: consecutive serial sections of retina were immunostained with MerTK (detected with anti-rabbit IgG-Alexa 594) and anti-S-cone opsin (detected with anti-rabbit IgG-Alexa 488), and the two patterns aligned. In all experiments, 4,6-di-amino-phenyl-indolamine was added during the second incubation to stain nuclei.

Slides were washed thoroughly with PBS, then mounted in PBS:glycerol (1:1) and observed under a fluorescence microscope (Optiphot 2; Nikon, Melville, NY). Images were obtained by charge-coupled device (CCD) camera video capture linked to a dedicated personal computer containing image analysis software (BIA; Nikon).

### Statistics

All graphical analysis for phagocytosis was done with Excel software, and all statistical analysis was done with R and Statistica 8 software (StatSoft, Créteil, France) using an Analysis of Variance (ANOVA) test. When ANOVA indicated significant differences (p=0.05), a post hoc analysis (Tukey test) was performed.

## Results

**Figure 2:**
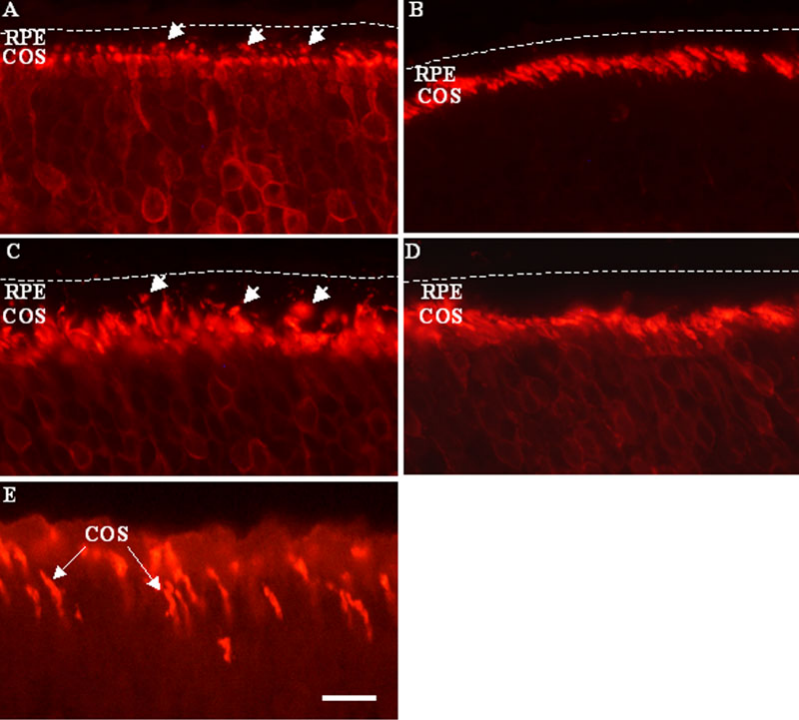
Immunohistochemical detection of rhythmic phagocytosis in short wavelength sensitive (S)-cones of young adult *Nrl^−/−^* and wild-type mice. **A** and **B**: These two sections were taken from animals killed under light-dark conditions at, respectively, hour 1 and hour 10 of zeitgeber time. **C** and **D**: These two sections were taken from animals killed under total darkness conditions at, respectively, hour 1 and hour 10 circadian time.. Sections immunolabeled with S-cone opsin antibody show the continuous row of cone outer segments across the section, and the presence of numerous immunoreactive inclusions (phagosomes=Ph) within the retinal pigment epithelium (RPE; *short arrows*) at ZT1 and CT1, but not at ZT10 and CT10. **E**: S-cone opsin immunostaining of wild-type mouse retina (ZT6) shows the presence of occasional labeled COS (long arrows). RPE, retinal pigment epithelium (the approximate level of the basal surface of the RPE) is shown by a dashed line in panels **A**-**D**. Scale bar in **E** represents 10 μm for all panels.

Figure 2 shows a representative image of *Nrl*^−/−^ mouse retinal sections stained with anti S-cone antibody. Heavy staining of the cone OS (COS) region is seen, corresponding to concentration of S-cone pigment in this cellular compartment. In sections made from mice killed at ZT1 under LD conditions, there are scattered discrete fluorescent dots visible within the pigmented band of RPE (arrows, Figure 2A). In sections made at ZT19, COS staining is unchanged, but there are fewer fluorescent inclusions seen in the RPE (Figure 2B). Similarly, in sections made from mice maintained in DD and killed at CT1, there are also immunopositive spots in the RPE (arrows, Figure 2C), whereas retinal sections from mice killed at CT19 contain fewer such profiles within the RPE layer (Figure 2D). Immunostaining of wild-type mouse retinas with antiS-cone opsin shows the occasional labeled COS (Figure 2E).

**Figure 3:**
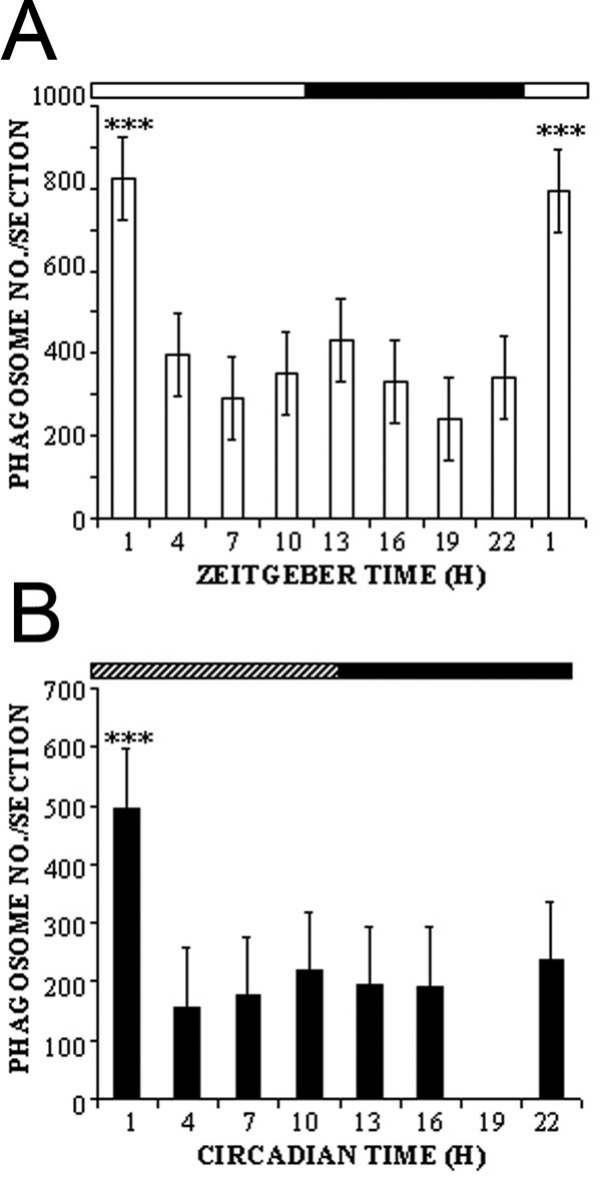
Cone shedding in *Nrl^−/−^* mice shows rhythmic activity in both cyclic light and constant darkness. Phagosome counts were made made every 3 h throughout both the light and dark periods under light-dark (LD) conditions (**A**) and through subjective light and dark periods under constant darkness (DD) conditions (**B**). A burst in the number of cone phagosomes was seen at ZT1 (LD) and at circadian time (CT1; total darkness), significantly different from all other values (***p<0.001). Phagosome numbers were lowest in late daytime.

Quantification of fluorescent inclusions within the RPE layer showed marked fluctuations as a function of time of day. Under LD conditions, there was a distinct peak at ZT1, with phagosome numbers between 2 and fourfold higher than at all other time points (Figure 3A). This value was highly significantly different from all other time points in the day or night. Phagosome numbers fell quickly to baseline levels, to roughly 50% lower at ZT4 and 70% lower by ZT7. There was a tendency toward a smaller second peak at ZT13 (1 h after lights-off), but this did not attain statistical significance. Thereafter, phagosome numbers were minimal (30%–40% the values at ZT1), before rising steeply again following the night-day transition. A very similar temporal profile was seen when *Nrl*^−/−^ mice were maintained under DD conditions for 36 h before sampling. Although the amplitude of phagocytosis was reduced by approximately 40%–50% at all time points, the shape matched closely that seen in LD, with a pronounced peak at CT1, some 2-4 fold higher than other points (Figure 3B).

**Figure 4:**
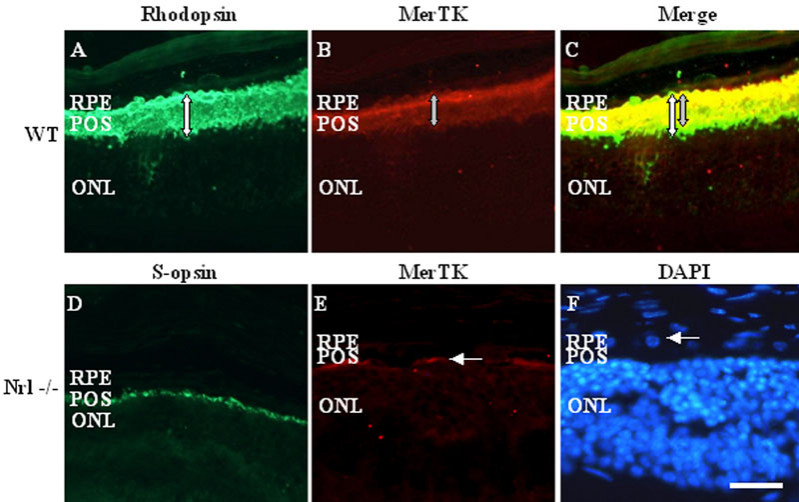
Expression of MerTK in retinal pigmented epithelium (RPE) of wild-type and *Nrl^−/−^* mice. In wild-type mice, the photoreceptor outer segment (POS) is intensely stained by rhodopsin antibody (**A**), and MerTK overlaps to a large extent with this stain (**B**). However, the MerTK label does not extend all the way to the base of the photoreceptor OS, so the merged image (**C**) shows a mixture of green (overlapping stain) and green (rhodopsin only). This is further illustrated by the two arrows (white to show the width of the rhodopsin stain, gray to show the width of the MerTK stain). S-cone opsin immunostaining in *Nrl^−/−^ retinas* shows aligned short POS (**D**), and the pattern of MerTK expression in *Nrl^−/−^* RPE is much reduced, compared to wild-type retinas, appearing as a discontinuous thin band running along the interface between the RPE and outer nuclear layer (ONL; arrow in **E**). **F**: The general structure of the *Nrl^−/−^* retina is seen by 4,6-di-amino-phenyl-indolamine (DAPI) staining of nuclei, showing the monolayer of rounded nuclei within the RPE (arrow). Scale bar represents 10 µm.

To see whether *Nrl*^−/−^ mice expressed the MerTK receptor, we also performed immunolabeling of wild-type and knockout mice retinal sections with a specific MerTK antibody. Wild-type mouse retinas underwent double immunolabeling against rhodopsin and MerTK. The rod OS were intensely immunolabeled with anti-rhodopsin antibody (Figure 4A). The MerTK immunostaining pattern was similar, with staining of the sub-retinal space corresponding to the apical microvilli of the RPE (Figure 4B). Merged images showed that the MerTK distribution extended almost the length of the OS (Figure 4C). In *Nrl*^−/−^ mice retinas, as before, S-cone opsin staining was prominent in the shortened cone OS (Figure 4D). MerTK was localized to the interface between the RPE monolayer and outer nuclear layer (ONL), the staining being limited to a narrow band and not extending into the sub-retinal space as in the wild-type retina (Figure 4E). The general morphology of the posterior pole in *Nrl*^−/−^ mice is shown in Figure 4F.

To see whether the overall phagocytic activity of blue cones in *Nrl*^−/−^ mice resembled values for “normal” rods and cones from other rodent species and strains under similar conditions, we calculated the number of phagosomes (rods and cones) seen at peak amplitude (ZT1/CT1), and normalized this to the number of rod- or cone-cell bodies within the same region. To reduce variability from the use of different techniques, we analyzed samples from normal wild-type mice, laboratory Wistar rats, and the diurnal rodent *Arvicanthis ansorgei*, all assayed with our immunohistochemical procedure. We also included data from published reports on two other species in which cat and chicken cone phagocytosis has been examined:. Table 1 expresses these values per 100 cell bodies. In four of the five species examined, peak cone activity was quite similar (8–12 phagosomes/100 cones); only in the cat were numbers considerably higher (20 phagosomes/100 cells). Rod phagosome frequencies in normal mice and rats were very close to the values for cone phagosomes in *Nrl*^−/−^ mice, whereas rod activity was much higher in *Arvicanthis* (38 phagosomes/100 rods).

**Table 1:** Comparison of phagosome numbers between rods and cones and between different species.

**Species**	**Peak cone value**	**Peak rod value**	**References**
cat	~20 per 100 cones	-	[25]
chick	~12 per 100 cones	-	[21]
Arvicanthis	~9 per 100 cones	~38 per 100 rods	Present study
Wistar rat	-	~7 par 100 rods	Present study
C57Bl6	-	~6 par 100 rods	Present study
*Nrl*^−/−^	~8 per 100 cones	-	Present study

To compare numbers between different retinas, these were calculated as a fraction of 100 cell bodies, either from our own data (rats, mice, *Arvicanthis*) or from previously cited information (cat, chicken). The percentage of phagosomes is quite similar between most of the different examples, except the elevated rod phagosome number in *Arvicanthis*.

## Discussion

We have used the all-cone *Nrl*^−/−^ mouse to document the temporal profile of cone phagocytosis under two lighting conditions—standard cyclic light and permanent darkness. The data show that mouse S cone-like photoreceptors, like rods, exhibit a daily rhythm in OS debris shedding/uptake by the RPE, and that this process shows a marked peak in activity shortly after light onset.

Cyclic light and/or circadian-driven rhythms in photoreceptor OS phagocytosis have been extensively explored in many species. Peak rod phagocytosis seems to be invariable, and occurs shortly after light onset in every species (nocturnal and diurnal) that has been studied, (e.g., chickens [21], mice [22], rats [23], ground squirrels [24], cats [25], and rhesus monkeys [26]). Data on cone phagocytosis are less abundant but show more irregularity. Most studies show maximal cone shedding in the night, as in diurnal chickens [21], fence lizards [27], tree squirrels [28], ground squirrels [24], and rhesus monkeys [26]. Some studies show the maximal cone peak at the beginning of the day, both in nocturnal species such as cats [25] and in diurnal species such as *Tupaia* [29] and *Arvicanthis ansorgei* [17,30]. Since mammals contain, in the great majority, mid/long wavelength cones [4,31], these studies have not provided information on the numerically minor S cones. Indeed, before the present investigation, it was not even known whether S cones underwent phagocytic turnover. The *Nrl*^−/−^ mouse thus represents a unique opportunity to analyze this population. It should be stated that cone identity in such mutant mice may not be strictly normal, and that the retina of this strain undergoes partial degeneration at an early age [14]. However, by a range of structural and functional criteria, they appear to be true cones [14,15].

Our results show that in *Nrl*^−/−^ mice, rhythmic phagocytosis occurs under normal cyclic conditions, where it may be regulated by light and/or an endogenous circadian clock. Under conditions of constant darkness, rod shedding continues with a profile similar to that of cyclic light [22,23,30,32]. Data for cone phagocytosis under constant darkness are scarce. In the cone-dominated retinas of fence lizards, rhythmic shedding continued when animals were placed in prolonged darkness, and the amplitude of shedding was greatly reduced [27], as seen in the present study. In our own studies on the diurnal rodent, *Arvicanthis ansorgei*, cone shedding also continued with the same profile in DD as in LD, but overall activity was actually enhanced by about 50% [30]. In this latter case, only mid/long wavelength cones were studied, and so the contrasting results may have reflected the altered responses of the two spectral types. But rod shedding also shows variable responses to prolonged darkness in terms of amplitude, with some studies showing increased activity [30,33] and others showing decreased activity [32]. Regardless, the continued cyclic shedding during constant darkness demonstrates the functioning of an endogenous circadian clock, capable of generating rhythmic behavior in the absence of light cues. This clock is able to synchronize peak shedding to the approximate beginning of day, at least after a single 24 h period of complete darkness.

Some observations on S-cone shedding as a function of lighting conditions deserve comment. According to our observations in wild-type mice, rats, and *Arvicanthis ansorgei,* and using previously published data from cats [25] and chickens [21], the relative maximal rate of phagocytosis is overall quite similar between rods and cones, and between species. These values are approximate, given that our data collection points may not have coincided precisely with actual peak activity, that we did not take into account differences in cell body or OS size, and that actual turnover rates for rods and cones are not known for all these species. But given the reduced cell density of ~80%, compared to the wild type [14], the length of OS [14] and RPE microvilli (~5–7 µm in wild-type: [20]; ~3 µm in *Nrl^−/−^*: [14]) in *Nrl*^−/−^ retinas, it is perhaps surprising that the phagocytic amplitude is so high (comparable to that of rods in normal mice). This is different from *Arvicanthis*, in which there is a clear difference in the turnover rates of rods and cones. Cone phagocytosis cannot be quantified in wild-type mouse retinas, because of the low cone numbers (3%–4%) [12] and diffuse distribution. Furthermore, rods are absent from the *Nrl*^−/−^ retina, so we are unable to compare data between the two strains. For the moment, we can only speculate that phagocytosis rates are perhaps related to relatively high turnover in S cones, or to a particular feature of the mutant retina (e.g., ongoing degeneration). Finally, the variability between species in terms of daily cone phagocytosis profiles, with in some cases an evening peak [21,24,26-28], and in others a morning peak overlapping with rods [25,29,30], makes it difficult to predict what the corresponding temporal cone profile would be in normal mice. The question is important, since two theoretical scenarios are possible: (1) normal mice have an evening cone peak (similar to most species studied), in which case the developmental switch from rods to cones in the *Nrl*^−/−^ mouse leaves the S cone-like cells with a partial functional rod phenotype; or (2) normal mice also have a morning cone peak, and the *Nrl*^−/−^ strain has a more fully cone-like functional phenotype.

Although the level of expression is reduced compared to wild-type mouse eyes, MerTK is clearly present at the apical RPE surface in *Nrl*^−/−^ mice, where it presumably participates in the binding and uptake of shed cone membranes, as in rats [7] and wild-type mice [34]. The distribution of the staining patterns for MerTK and S-cone opsin, showing shortened microvilli and OS respectively, suggests that RPE/OS interactions must be less intimate than in wild-type retinas.

In summary, the *Nrl*^−/−^ mouse presents a unique opportunity to examine cone shedding (moreover, blue cone shedding) in the otherwise highly rod-dominant mouse retina, and represents a valuable model to investigate the molecular mechanisms involved in cone recycling.
